# The E3 Ligase CHIP: Insights into Its Structure and Regulation

**DOI:** 10.1155/2014/918183

**Published:** 2014-04-24

**Authors:** Indranil Paul, Mrinal K. Ghosh

**Affiliations:** Cancer Biology and Inflammatory Disorder Division, Council of Scientific and Industrial Research-Indian Institute of Chemical Biology (CSIR-IICB), 4 Raja S.C. Mullick Road, Kolkata 700032, India

## Abstract

The carboxy-terminus of Hsc70 interacting protein (CHIP) is a cochaperone E3 ligase containing three tandem repeats of tetratricopeptide (TPR) motifs and a C-terminal U-box domain separated by a charged coiled-coil region. CHIP is known to function as a central quality control E3 ligase and regulates several proteins involved in a myriad of physiological and pathological processes. Recent studies have highlighted varied regulatory mechanisms operating on the activity of CHIP which is crucial for cellular homeostasis. In this review article, we give a concise account of our current knowledge on the biochemistry and regulation of CHIP.

## 1. Introduction

Maintaining the integrity of the proteome is essential for normal cellular functions. Although the native state of a protein is energetically favoured, the attainment of it is a thermodynamic challenge. The matured form of a given protein remains in a dubious equilibrium, often misfolding to various degrees during its lifespan, as a result of stochastic fluctuations, presence of destabilizing mutations, stress conditions, or unique metabolic challenges, such as cancer or aging. In addition, a myriad of posttranslational modifications (such as phosphorylation and acetylation) can mark native proteins for regulated degradation borne out of signaling necessities [1].

The carboxyl-terminus of Hsc70 interacting protein or CHIP (referred to hereafter as CHIP) is a U-box type chaperone associated E3 ligase. CHIP was identified in an interaction-cloning screen as a TPR-containing and Hsc/p70 interacting protein. It was found to be highly expressed in tissues (such as skeletal muscle) with high metabolic activity and protein turnover [2]. This dual-function cochaperone/E3 ligase protein activates HSF1 under various stress conditions and confers protection against apoptosis. The presence of CHIP is essential for eliciting a normal heat stress response [3, 4]. In contrast to cochaperones Hip, Hsp40, and BAG1 which promote substrate binding and release, CHIP was found to attenuate ATP hydrolysis and inhibit the forward Hsc70/Hsp70 cycle, at least* in vitro* [2, 5]. Predictive of its U-box, CHIP was identified as the first chaperone associated E3 ligase that targets Hsp90 (partially folded) and Hsp70 (misfolded) clients for degradation through the 26S proteasome [6–9] and is suggested to be also involved in substrate delivery to the proteasome [6, 10].

Over the last decade, CHIP has been demonstrated to regulate a number of biological processes. Aberrations in protein expression and activity of CHIP are observed in many pathological conditions stressing the need to understand its regulation. In this review we attempt to comprehensively summarize the recent findings on CHIP, focusing mostly on its structure and various regulatory mechanisms operating on it. Finally, we evoke some important questions of CHIP biology.

## 2. Initial Discovery and Characterization

Tetratricopeptide repeats (TPR) are protein-protein interacting motifs that consist of loosely conserved 34-amino acid stretch usually present in tandem in proteins with diverse cellular functions [11], particularly those that interact with chaperones including Hip, Hop, and the cyclophilins [12–14].

**Figure 1:**
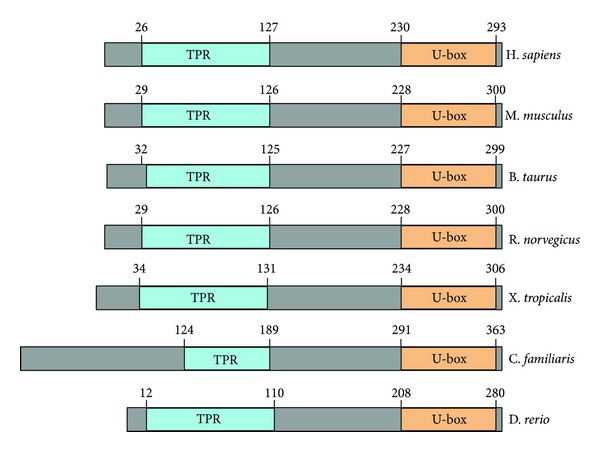
Schematic diagram showing alignment of CHIP from different species. The figure clearly depicts very high conservation in the central and C-terminal regions. The N-terminal protein-protein interaction motif is apparently amenable to alterations, probably for species specific specialization of interaction.

In an attempt to identify novel TPR-containing proteins in the human heart a fragment corresponding to nucleotides 721 to 1150 of the human CyP-40 (cytochrome P-40) cDNA was radiolabeled with [*α*-^32^P] dCTP and used to screen a phage library of human heart cDNA at low stringency. CHIP was identified through this screen [2]. CHIP cDNA encodes a 34.5-kDa protein. Evolutionary, CHIP is a well-conserved protein with an amino acid sequence similarity of ~98% with mouse and ~60% with the fruit fly [11]. The C-terminus 94 residues (the U-box region) seem to be least altered with ~87% similarity among these species' (Figure 1). Intracellularly, CHIP was found to primarily localize to the cytoplasm under quiescent conditions [2] although a fraction of CHIP was later found to be present in the nucleus as well [10].

### 2.1. CHIP: A Protein Quality Control E3 Ligase

The classical role of chaperones was regarded as those of folding and salvaging proteins. However, it was apparently clear that each and every newly synthesized polypeptide that engages with chaperones for its folding could not reach the native state, and thus there must be a link between the chaperones and the degradation pathways. CHIP provided that link. CHIP was identified as a TPR-containing protein that inhibited forward cycle of chaperones. However, the curtain on the actual role of CHIP was raised when it was shown to have intrinsic E3 ligase activity owing to the C-terminus U-box, a domain related to the well-characterized RING-finger. Experimental studies with increased cellular levels of CHIP found a marked shift towards degradation of the Hsp70/Hsp90 clients, glucocorticoid receptor (GR) [6, 15], and CFTR [10, 16]. Definitive evidence for a quality control role of CHIP was provided when it was shown to selectively ubiquitinate thermally denatured luciferase (and not the native form) when captured by Hsc70 and Hsp90 [6, 8, 17]. It can be noted here that in bacteria, which lack ubiquitination, ATP-dependent proteolysis also depends on chaperones for substrate recognition by Lon proteases and ClpP, as do yeasts, which are known to have CHIP. In other words, chaperones are an excellent way to identify misfolded proteins.

That CHIP may have participation in protein turnover was hinted by initial observations depicting a relatively higher expression of CHIP mRNA in tissues with a large proportion of terminally differentiated, nonproliferating cells and high levels of metabolic activity such as skeletal muscle, heart, and brain [2]. The physiological importance of CHIP came into light with the observations that ~20% of CHIP null (CHIP − /−) mice die at embryonic stages and 100% fail to survive thermal stress [18].

In mammals, CHIP is located within chromosome 16. No isoforms or transcript variants have been reported yet for CHIP.

## 3. Structural Organization of CHIP

### 3.1. Primary Structure of CHIP

The primary organization of CHIP's structure comprises two structured motifs: an N-terminus TPR domain and a C-terminus U-box domain separated by a central coiled-coil region [2, 19]. The entire TPR domain (residues 26–131) consists of three pairs of TPR repeats where each TPR consists of two antiparallel *α*-helices separated by a turn, such that apposed bulky and small side chains form a “knob and hole” structure. The hydrophobic surface of this structure imparts the ability of protein-protein interaction to the TPR motifs [20, 21]. The N-terminus of an elongated seventh helix (helix 7) packs against the third TPR repeat. The structure of this helix 7 has significant consequences for CHIP (see below). U-boxes are structurally related to RING-finger domains, except that U-boxes are stabilized by hydrogen bonds instead of zinc binding [22]. Mechanistically, there are two categories of E3 enzymes [23]. HECT domain E3 ligases form transient thioester linkages with ubiquitin to a C-terminus active-site cysteine residue and transfer the ubiquitin moiety to the substrate bound to the N-terminus. On the other hand, RING-finger or U-box E3 ligases simply act as scaffolds or adaptors, which position the substrate in precise proximity to the E2-ubiquitin thioester (E2-Ub). In CHIP, the U-box domain (residues 232–298) consists of a pair of *β*-hairpins running into a short *α*-helix, followed by a third hairpin leading to a C-terminal *α*-helix. The central region (residues 128–229) forms helical hairpins and has been shown to be essential for dimerization of CHIP and consequently its function [19].

### 3.2. Structural Aspects of CHIP Activity

Another interesting structural feature of CHIP is that it forms an asymmetric homodimer, meaning that each protomer inherently adopts significantly different conformations in the dimer. The structural explanation is as follows. First, CHIP dimerization involves two interacting interfaces between each monomer, the U-box domain and the region linking the TPR and U-box. The U-box forms a parallel dimer with a ~2-fold symmetry. Similarly, the core of second interface, the helical hairpins, also displays ~2-fold symmetry. However, the local 2-fold axis relating the respective symmetry-axes of U-box and helical hairpins does not coincide but is tilted by 30°. Second, helix 7 has a different structure in the two protomers of the CHIP dimer. In one, it is a straight *α*-helix while in the other the polypeptide is broken into two separate and mutually perpendicular *α*-helices. The functional consequence of these arrangements is that only one of the U-boxes in the CHIP dimer is active thus effectively displaying a “half-of-sites” activity which allows it to form monotonic polyubiquitin chains [24].

### 3.3. Interaction with E2s

CHIP was previously shown to cooperate with the UbcH5 family of E2s, to catalyze Lys-48-linked polyubiquitination. UbcH5 is a stress associated E2. UbcH5~Ub conjugates have been shown to adopt both infinite spiral and linear staggered arrangement (backside interaction). Interestingly, CHIP can directly interact with four (or more) UbcH5~Ub conjugates allowing wide conformational flexibility during polyubiquitination of substrates [25]. One consequence of this is the possibility of formation of forked ubiquitin chains (see Section 4.3.10).

Later, Xu and coworkers [26] reported CHIP to interact with the dimeric ubiquitin E2 complex Ubc13-Uev1A, which catalyzes the synthesis of Lys-63-linked polyubiquitination. They analyzed crystal structures of mouse CHIP U-box in complex with Ubc13-Uev1a and found a common “Ser-Pro-Ala” motif present in UbcH4, UbcH5, and Ubc13 that mediates, and is necessary for, their interaction with the CHIP U-box. Although the catalysis of K63-linked polyubiquitination is an inherent structural feature of Ubc13-Uev1A, it is not clear at present how the binding of CHIP to Ubc13-Uev1A facilitates the process. Interestingly, CHIP only stimulates the formation of free K63-polyubiquitin by Ubc13-Uev1a and thus may have to interact sequentially with other E2 enzymes to attach K63-linked polyubiquitin chains on substrates. CHIP binds 3- to 5-fold more strongly to uncharged Ubc13 than UbcH5a. It remains to be seen whether CHIP displays similar relative binding affinities towards ubiquitin charged E2s [26].

The physiological ramifications of the fact that CHIP can function with different E2s to catalyze distinct forms of polyubiquitination are yet to be studied in detail.

## 4. Regulation of CHIP

### 4.1. CHIP Is Regulated at the Transcriptional and Posttranscriptional Levels

#### 4.1.1. Transcriptional Regulation

**Figure 2:**
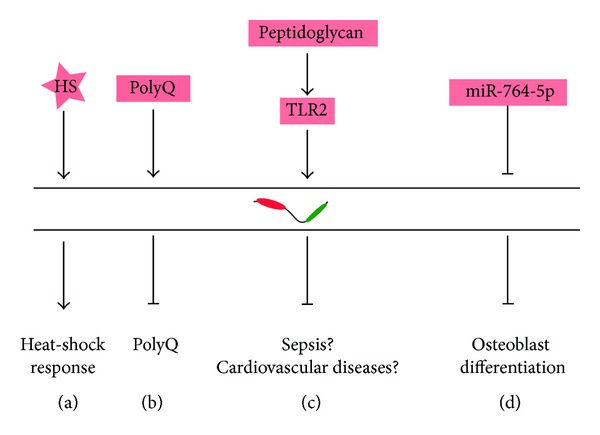
Schematic representation of known transcriptional and posttranscriptional regulation of CHIP under various conditions.* See text for details*.

A limited number of studies have been devoted to understand the regulation of CHIP mRNA under different physiological and pathological contexts. Under normal conditions, the basal levels of Hsc70, Hsp90, and its cochaperones suffice to maintain protein homeostasis. However, it is not difficult to imagine that a massive build-up of misfolded polypeptides resulting from cellular stresses would need rapid readjustments in the cellular levels of these proteins. Indeed, the mRNA levels of Hsp70 and/or CHIP are upregulated under various stress conditions such as heat-shock, overexpression of the pathogenic form of polyQ [27], and oxidative damage [28] and have been shown* in vivo* or* in vitro* to provide protection (Figures 2(a) and 2(b)) [29, 30]. From a clinical point of view, such stressors are an integral feature of various human pathologies including neurodegenerative disorders, muscular dystrophies, and heart ailments.

Recently, CHIP has been intimately linked to various inflammatory responses such as regulation of IL-4 and TLR signaling, T cell activation, and DALIS formation [31–36]. Treatment of RAW264.7 cells by peptidoglycan activates TLR2 receptors and through JNK pathway upregulates expression of CHIP. Interestingly, although CHIP is crucial for proper TLR2/4/7/9 signaling, its expression seems to be controlled by only TLR2 signaling as LPS (TLR4 ligand) or CpG ODN (TLR9 ligand) does not stimulate endogenous CHIP overexpression [37, 38] (Figure 2(c)).

The expression of CHIP is also altered in human malignancies. Both mRNA and protein levels of CHIP have been found to be lower than corresponding normal tissue in breast [38, 39], colorectal [40, 41], and gastric cancer [42] and correlate highly with tumour prognosis.

#### 4.1.2. Posttranscriptional Regulation

An instance of posttranscriptional regulation of CHIP mRNA has been reported in the context of bone morphogenesis. CHIP is downregulated in calvarial and osteoblast progenitor cells during osteoblast differentiation. In MC3T3-E1 cells, the microRNA miR-764-5p has been shown to inhibit the translation of CHIP mRNA by binding at its 3′-UTR. Perturbations of miR-764-5p could be rescued by a concomitant and opposite extraneous expression of CHIP. Translational repression of CHIP mRNA by miR-764-5p was found to be essential for proper osteoblast differentiation (Figure 2(d)) [43]. Clearly, regulation of CHIP at the mRNA level is an important strategy for regulating its biological activity.

### 4.2. Posttranslational Modifications of CHIP Are a Potential Mode of Regulation

**Figure 3:**
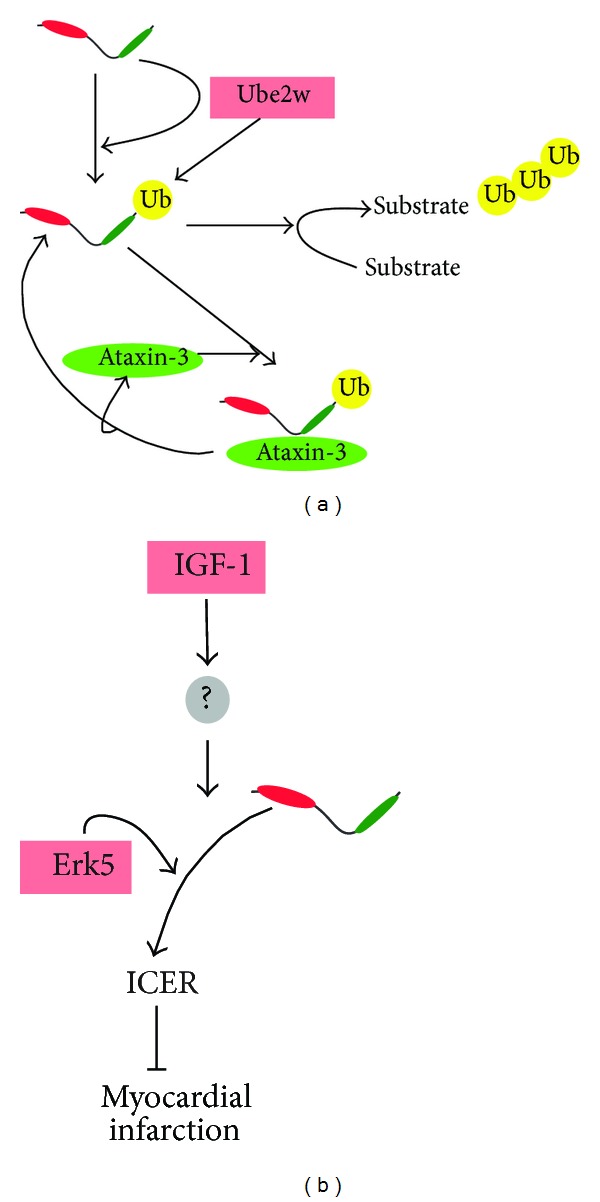
Schematic representation of known posttranslational modes of regulation of CHIP. (a) Monoubiquitination; (b) phosphorylation.* See text for details*.

The activity of CHIP is also regulated through posttranslational modifications [44]. Such modifications of CHIP likely dictate recruitment of cofactors which in turn may participate in restricting the type and length of ubiquitin chains produced [45, 46]. CHIP is known to undergo regulatory ubiquitination in cells and* in vitro* [8, 47, 48], which facilitates targeting of its substrates for proteasomal degradation [5]. For example, the proteasomal subunit S5a is a ubiquitin-interacting motif (UIM) containing protein which stimulates turnover of CHIP substrates by preventing the formation of forked ubiquitin chains [49]. Interestingly, ubiquitination of CHIP increases under conditions in which CHIP levels and activity are increased [50]. The mechanistic details have been recently worked out [51]. Ataxin-3, a specialized ubiquitin-interacting motif (UIM) containing deubiquitinase (DUB), associates with CHIP monoubiquitinated by the initiator E2 Ube2w (and perhaps other E2s) at Lys 2 of CHIP [51]. This modification occurs before or just after polyubiquitin chain initiation. Consequently, ataxin-3 provides a chain editing activity effectively determining the dynamics of substrate ubiquitination by CHIP. Upon completion of substrate ubiquitination, as determined by chain length, ataxin-3 presumably binds ubiquitinated substrate through its UIMs and deubiquitinates CHIP, thus terminating the reaction (Figure 3(a)) [51].

**Figure 4:**
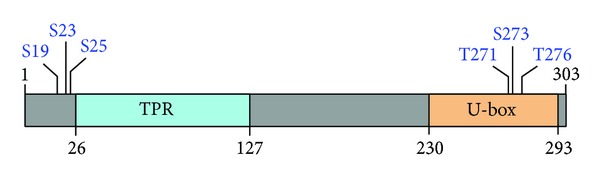
Schematic representation of various conserved putative phosphorylation sites on CHIP (source: http://www.hprd.org/).* See text for details*.

Although direct evidence showing similar posttranslational modifications other than ubiquitination in regulating the activity of CHIP is still lacking, the possibility of such events cannot be ruled out. Indeed, the N- and C-terminal regions of CHIP have been proposed to contain functional phosphorylation sites (Figure 4) [52]. In an interesting study, an association of the phosphatase laforin and the E3 ligase malin with CHIP has been shown to be essential to achieve cellular heat-shock response mediated through HSF1. Either laforin or malin does not affect the nuclear localization, hyperphosphorylation or the trimerization property of HSF1. Although the nuclear translocation of CHIP seems to be promoted by the complex [53], what effects does the complex have on the phosphorylation status of CHIP has not been reported yet but presents an interesting possibility.

Associations between CHIP and kinases such as ERK5 and Lim kinase 1 (LIMK1) have also been reported [54, 55]. The ERK5-CHIP interaction was shown to be an essential module operating downstream of IGF-1 induction having a cardioprotective effect mediated through inducible cAMP (cyclic adenosine monophosphate) early repressor (ICER) destabilization. ERK5 activation leads to increased CHIP ubiquitin ligase activity possibly through a conformational change in CHIP [55] (Figure 3(b)). LIMK1 has been shown to associate with CHIP in a complex of which Parkin is also a member [54]. Parkin ubiquitinated LIMK1 in dopaminergic neuronal BE(2)-M17 cells but not in kidney-derived HEK293 cells and attenuated LIMK1-induced cofilin phosphorylation and subsequent accumulation of actin filaments. Conversely, LIMK1 attenuated Parkin autoubiquitination and its activity in a phosphorylation independent manner [54]. In both of these cases, the possibility of a phosphorylation dependent regulation of the activity of CHIP seems to be an interesting possibility and needs further investigation.

#### 4.2.1. Half-Life

Other than the posttranslational modifications that directly affect the function of CHIP, the half-life or protein stability of CHIP may also be a possible mode of its regulation. For instance, a study specifically devoted to this aspect of CHIP found that the TPR domain of CHIP when isolated was monomeric and stable, while its U-box domain formed dimers and had very low stability [56]. Thus, more biochemical studies are clearly needed to shed light on this important aspect of CHIP biology.

### 4.3. Activity of CHIP Is Regulated by Interactions with Other Proteins

A number of proteins have been shown to interact with CHIP and regulate its activity.

#### 4.3.1. Ca^2+^/S100 Proteins

**Figure 5:**
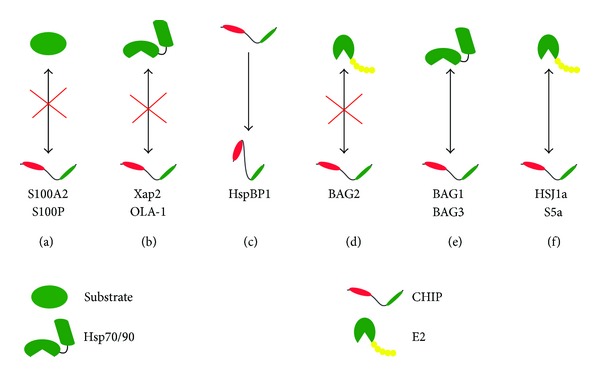
Schematic representation of regulatory protein interactions between CHIP and various reported proteins. (a) Competition with substrate binding, (b) competition with chaperone binding, (c) conformational modification of CHIP, (d) disruption of CHIP and E2 interaction, (e) facilitation of chaperone binding, and (f) facilitation of interaction with E2.* See text for details*.

S100 proteins are a family of at least 25 low molecular weight multifunctional members found in vertebrates, with regulatory roles in a variety of cellular processes [57, 58]. Recently, S100A2 and S100P were found to associate* in vitro* and* in vivo* (Huh-7 and Hep3B cells) with TPR-containing cochaperones, CHIP (and also Hop), in a Ca^2+^ dependent manner. This interaction seems to compete with substrate binding and consequently suppress the E3 ligase activity of CHIP towards Hsp70, Hsp90, p53, HSF1, and Smad1. Interestingly, S100 has no effect on CHIP-UbcH5a interaction. Further, Lys-30 and Pro-269 were found to contribute to S100-CHIP interaction. This report identifies a novel mechanism of protein quality control in response to intracellular Ca^2+^ signaling (Figure 5(a)) [59].

#### 4.3.2. Xap2

The hepatitis B virus X-associated protein (Xap2) is a TPR-containing protein with poorly defined functions, except that it plays a role in dioxin receptor (DR) metabolism. Along with Hsp90 and p23, Xap2 retains the DR in a latent state until it is activated upon binding of dioxins or structurally related forms of xenobiotics and translocates to the nucleus for activating transcription of target genes involved in xenobiotic metabolism [60–62]. CHIP has been proposed to degrade DR efficiently in the nucleus, an activity that can be overcome by overexpression of XAP2. Apparently XAP2, which also contains TPR domain, competes with CHIP for binding with Hsp90, thus shifting the balance of the triage towards folding (Figure 5(b)) [48].

#### 4.3.3. OLA-1

Obg-like ATPase 1 or OLA-1 is a highly conserved ~45 kDa cytosolic ATPase and belongs to the TRAFAC class, Obg family, and YchF subfamily of P-loop NTPases. YchF subfamily of NTPases binds and hydrolyzes ATP more efficiently than GTP [63]. Recently, OLA-1 was shown to positively regulate the heat-shock response by protecting Hsp70 from CHIP mediated degradation. The half-life of Hsp70 is shortened in OLA-1−/− MEFs. OLA-1 competes with CHIP for binding at the C-terminal of Hsp70 and thus precludes CHIP activity towards Hsp70 (Figure 5(b)) [64].

#### 4.3.4. HspBP1

It is a nucleotide release factor of Hsc70 that has similar expression patterns as of CHIP and competes with BAG1 and BAG2 for the ATPase domain of Hsc70 [65]. In HeLa cells, HspBP1 apparently inhibits the ubiquitin ligase activity of CHIP bound to Hsc70, but not Hsp90, in a noncompetitive manner. HspBP1 presumably induces conformational changes of the chaperone complex that keep ubiquitin acceptor sites of the substrate from the reach of CHIP. HspBP1 itself is not recognized as a substrate protein by CHIP. Interestingly, although overexpression of HspBP1 influenced CFTR biogenesis, it did not have any effect on glucocorticoid hormone receptor turnover, pointing towards a yet poorly understood mechanism of client specificity (Figure 5(c)) [16].

#### 4.3.5. BAG1

Human Bcl-2-associated athanogen (BAG) is a family of six known members (BAG1 to BAG6) with diverse cellular functions [66–68]. They are characterized by the presence of a BAG domain implicated in direct interaction with the ATPase domain of Hsc/p70 and can regulate chaperone activity in both positive and negative manners [69]. During the sorting of chaperone clients to the proteasome, CHIP can cooperate with the Hsc70 cochaperone BAG1. The two cochaperones interact simultaneously with Hsc70, as BAG1 binds to the amino terminal ATPase domain of the chaperone and leaves the carboxy-terminus of Hsc70 available for an association with CHIP [7, 70]. BAG1 is intimately involved in the regulation of the ATP-dependent peptide binding and release cycle of Hsc70 by stimulating nucleotide exchange [71, 72]. Furthermore, BAG1 associates with the proteasome through an integrated ubiquitin-like domain and thereby recruits Hsc70 chaperone complexes to the proteasome [73]. As a consequence BAG1 stimulates CHIP-induced degradation of the glucocorticoid hormone receptor (Figure 5(e)) [7].

#### 4.3.6. BAG2

It is a protein of ~34 kDa and exists as dimers under physiological conditions. Immunoprecipitates of overexpressed BAG2 from HeLa cells contained CHIP, which was released upon ATP treatment, a reflection of ATP-regulated chaperone/cochaperone interactions. BAG2 inhibits the ubiquitin ligase activity of CHIP bound to Hsc70 by disrupting the interaction between CHIP and its E2 and UbcH5b. BAG2 itself is not ubiquitinated by CHIP (Figure 5(d)) [74, 75].

#### 4.3.7. BAG3

There is a functional reciprocality between BAG1 and BAG3 during conditions of acute stress or aging, often referred to as the “BAG1-BAG3 switch.” BAG3 has recently gained much popularity as a mediator of a novel macroautophagy pathway, termed chaperone-assisted selective autophagy or CASA that exploits Hsp70 and cochaperones including CHIP. Both BAG3 and CHIP have been found to induce the association of the phagophore-interacting ubiquitin adaptor p62 with Hsp70. P62 acts as an adaptor between ubiquitinated substrates and phagophore membranes. BAG3 also indirectly stimulates the binding of CHIP to Hsp70 complex. Reciprocally, CHIP ubiquitinates BAG3 further facilitating its interaction with p62 eventually resulting in engulfment by phagophore (Figure 5(e)) [76, 77].

#### 4.3.8. BAG5

BAG5 protein was shown to interact with CHIP through Hsp70 and inhibit the E3 ubiquitin ligase activity of CHIP towards *α*-synuclein. The mechanism by which BAG5 inhibits CHIP remains to be elucidated [75, 78].

#### 4.3.9. HSJ1a

Human HSJ1 isoforms, HSJ1a and HSJ1b, are cochaperones which facilitate forward cycle of Hsc70, protect polyubiquitin chains against trimming, and promote proteasomal sorting of ubiquitinated proteins [79, 80]. Coexpression of CHIP and HSJ1a in HeLa cells led to a significant increase in the amount of HSJ1a bound ubiquitinated polypeptides. HSJ1a directly stimulated CHIP mediated ubiquitination. Further, CHIP mediated ubiquitination of HSJ1a promoted client sorting to the proteasome machinery (Figure 5(f)) [81].

#### 4.3.10. S5a

Under* in vitro* conditions many ring-finger and U-box E3 enzymes including CHIP, in conjunction with UbcH5, have a tendency to form forked ubiquitin chains in which two Ubs are linked to adjacent lysines on the proximal Ub instead of standard isopeptide linkages in which the C-terminal carboxyl group of a Ub is coupled through an isopeptide bond to the *ε*-amino group on one of the 7 possible lysines on the proximal Ub. These forked linkages resist proteasomal isopeptidases and bind only weakly to the 26S proteasome compared to standard linkages [82]. In a study to identify cellular Ub-binding proteins that prevent formation of these nondegradable conjugates, S5a (Rpn10) was identified. S5a was found to enhance proteasomal degradation of firefly luciferase ubiquitinated by CHIP/UbcH5 by preventing forked chain formation. S5a occurs free in large amounts in the cytosol. Cells lacking S5a are viable but show defects in proteasomal degradation of a specific subset of cellular proteins (specifically where UbcH5 is the E2) resulting in increased levels of Ub conjugates, as often found in many neurodegenerative diseases. Interestingly, S5a gets ubiquitinated by CHIP/UbcH5 and seems to be essential for eliciting a proper heat-shock or oxidative stress response (Figure 5(f)) [49, 83].

#### 4.3.11. Ataxin-3

The deubiquitinase (DUB) ataxin-3 contains an amino-terminal protease domain followed by three UIMs that bind chains of four or more ubiquitins [84, 85]. Unlike other characterized DUBs that function to deubiquitinate substrates and rescue them from proteasomal delivery [86], ataxin-3 promotes the flux of substrates through degradation pathways [87, 88]. Recently, ataxin-3 has been shown as an example of a CHIP-associated DUB (Figure 3(a) and above for details). In pathological conditions such as spinocerebellar ataxia type 3 the polyQ-expanded ataxin-3 has higher affinity for CHIP and correlates with decreased levels of CHIP ultimately causing proteotoxicity [51].

### 4.4. Collaboration with Other E3's—An E4 Activity of CHIP

Apart from a direct ubiquitin ligase activity CHIP is also known to facilitate ubiquitination mediated through other E3's, in effect functioning as an E4 ligase. The defining example is Parkin. Parkin is a RING-finger type E3 ligase initially identified as the culprit (due to an inactivating mutation) in an autosomal recessive form of juvenile Parkinson's disease (AR-JP) [89]. Later, the Pael-R membrane receptor was found to be a substrate of Parkin and its accumulation in endoplasmic reticulum (ER) caused ER-stress induced neuronal death and hence the disease [90, 91]. In an interesting study CHIP was found to potentiate Parkin mediated Pael-R ubiquitination, both* in vivo* and* in vitro*. Surprisingly, Hsp70 bound Pael-R is a poor substrate of Parkin, and it is only when CHIP displaces Hsp70 (presumably by blocking its ATPase activity) from Parkin/Pael-R complex can Parkin fully exercise its E3 activity towards Pael-R. Although CHIP can bind to both Parkin and Pael-R, it showed no ubiquitin ligase activity for Pael-R and thus functions purely as an E4 ligase in this scenario [47, 92, 93].

Another E3 ligase with which CHIP has been found to interact is the E3 ligase complex SCF^Skp2^ (Skp1-Cullin-F box^S-phase  kinase  associated  protein 2^). Among many others, p27, c-Myc, E47, and Tal1/Scl (T cell acute leukemia 1/stem cell leukemia) are well known substrates of SCF^Skp2^ [94–98]. In the context of Tal1/Scl and E47, which forms heterodimers to activate transcription, the role of CHIP has been found to be crucial. Although both Tal1/Scl and E47 associate with Skp2 independently and are apparently ubiquitinated by SCF^Skp2^, overexpression of chaperone-noninteracting and E3 ligase deficient forms of CHIP or knockdown of endogenous CHIP diminishes Tal1/Scl and E47 ubiquitination. Interestingly, despite Skp2 interacting with Hsc70 and CHIP with cullin1, the association of CHIP with Hsc70 seems to be critical for efficient degradation. Thus, it has been suggested that CHIP could promote activity of some factors required for SCF^Skp2^ mediated ubiquitination of at least a few of its substrates in a manner dependent on chaperone binding and “E4” activity of CHIP [97].

### 4.5. Subcellular Localization of CHIP Is Regulated

Despite being initially characterized as a cytoplasmic protein, the nuclear existence of CHIP is now well established. Using techniques such as immunofluorescence microscopy (IFM), immunohistochemistry (IHC), and immunoblotting (IB) of cytoplasmic and nuclear fractions, CHIP has been shown to localize to the nucleus under different physiological contexts [50]. Consistently, we found that CHIP (both endogenous and exogenous) is expressed in both the cytoplasm and the nucleus of DBTRG-05MG [99], HEK293, C6, U87MG, U118MG, U138MG, MCF7, and HepG2 cells (unpublished).

Examples exist to support the notion that subcellular localization of CHIP is regulated and in turn can regulate its functional activity. Upon heat-shock of murine fibroblasts HSF1 and CHIP are known to rapidly translocate to the nucleus as a part of the response and reaccumulate in the cytoplasm during recovery from heat-shock [18]. In this context, malin and laforin have recently been shown to promote the nuclear translocation of CHIP and to be essential for providing a complete protection against heat-shock [53]. In rodent brain and primary cortical neurons, CHIP rapidly (within 5–10 minutes) and transiently (for up to 60 minutes) accumulated in the nucleus following heat-shock and oxygen-glucose deprivation which was linked to the capacity and capability of these cells to recover and survive [100]. Although the identities of nuclear substrates of CHIP are obscure, a few have nevertheless been reported. For instance, endogenous CHIP and mutant AR were found to colocalize in the nuclei of spinal anterior horn neurons of the AR-97Q mice and SBMA (spinal and bulbar muscular atrophy) patients [101]. Similarly, CHIP has been found to colocalize with ataxin-1 nuclear inclusions [102, 103]. Coimmunoprecipitation experiments using cytoplasmic and nuclear fractions of mammalian cells showed that CHIP interacted with p65 [104], RUNX1 [105], and RUNX2 [106] proteins only in the nuclear extracts but not from the cytoplasmic fractions. Furthermore, under heat-stress CHIP not only assisted p53 to regain its native and transcriptionally active form but also comigrated with it into the nucleus and was found tethered to p21 and p53 promoters along with p53, as revealed by chromatin immunoprecipitation [107].

In addition to the nucleus, studies have pointed towards a role for CHIP in the endoplasmic reticulum (ER). CHIP has been shown to colocalize with CFTR and Hsp70 at the ER membrane, indicating a role in CFTR biogenesis. Later, inactivation of CHIP was shown to permit a subpopulation of CFTR-ΔF508 to fold, escape the ER, and accumulate as a maturely glycosylated species [10, 108]. Growth hormone receptor (GHR) is efficiently folded in the ER and under conditions of CHIP depletion both precursor and mature GHR accumulate inside the cells [109]. Recently, a study linking ER stress and tauopathy reported that upon elicitation of ER stress (using glucose deprivation) the interaction between tau, a known target of CHIP, and CHIP significantly decreases leading to an accumulation of tau and consequent tauopathy [110].

However, the details of the molecular mechanisms that modulate the intercompartmental distribution of CHIP need to be further explored which might provide new insights into the significant role of CHIP inside the various organelles in regulating biological phenomena.

### 4.6. Substrate Recognition by CHIP Is Specific

The intimate functional association of CHIP with the chaperones has raised concerns regarding the specificity of CHIP towards its substrates. The suspicion that CHIP may promiscuously ubiquitinate any protein fulfilling the criteria of being structurally disorganized to be eligible for being recognized by the chaperones may not be correct. On the contrary several reports have documented varying degrees of dependency of auxiliary factors that potentially determine the response of CHIP towards its substrates. However, it should be mentioned that the extent of involvement of the chaperones in every such case was not determined conclusively.

#### 4.6.1. Posttranslational Modification (PTM) of Substrates

Phosphorylation is one of the most prolific PTMs in the cell. PKA (protein kinase A) mediated phosphorylation of HDAC8 (histone deacetylase 8), under normal and tumor conditions, recruits EST1B (ever shorter telomeres 1B) and Hsp70/Hsp90 in a complex in a manner which precludes CHIP from ubiquitinating EST1B, which is otherwise a substrate of CHIP [111].

In prostate cancers, hormone-ablation therapy, in most cases, leads to more aggressive androgen-refractory disease which is characterized by overactivated androgen receptor (AR) mediated signaling and accumulation of cells with neuroendocrine characteristics such as elevation of PTHrP (parathyroid hormone-related protein) levels. PTHrP activates EGFR and Src kinase (through an unknown mechanism) which in turn phosphorylates AR on Tyrosine 534. This phosphorylation event reduces the interaction between AR and CHIP leading to AR stabilization and enhanced growth of prostate cancer cells even at very low levels of androgen [112].

Similarly, under oxidative stress the protein kinase c-Abl phosphorylates another kinase MST1 (mammalian Ste20-like kinase 1) at Tyrosine 433 which in turn accomplishes two roles: first, the event inhibits the degradation of MST1 through CHIP and, second, triggers association between MST1 and FOXO3 (Forkhead box O3), thereby activating the MST1-FOXO signaling, leading to cell death in both primary culture neurons and rat hippocampal neurons [113].

Another case of regulated engagement of CHIP for substrate ubiquitination is provided by CYP2E1, a liver ER cytochrome P450, responsible for xenobiotic metabolism. CYP2E1 displays a biphasic turnover with respect to presence or absence of its substrates (low molecular weight xenobiotics, carcinogens, and endogenous ketones). Both UBC7/gp78 and UbcH5a/CHIP were found to ubiquitinate CYP2E1* in vitro*, a process blocked by intracellular inhibition of PKA or PKC. Several phosphorylation/ubiquitination clusters in CYP2E1 were also identified [114].

Apart from regulatory phosphorylation on substrates, the association of ERK5 and CHIP has been shown to be essential for CHIP mediated cardioprotection (see above). Specifically, diabetic mice subjected to myocardial infarction (MI) overexpress ICER protein which reduces antiapoptotic Bcl-2 levels thus leading to apoptosis. Under these conditions, IGF-1 (insulin-like growth factor 1) induction or MEK5*α* overexpression results in a specific and obligatory association between ERK5 and CHIP; this increases the E3 ligase activity of CHIP, through a hitherto unknown mechanism, subsequently degrading ICER and inhibiting apoptosis (Figure 3(b)) [55]. The importance of ERK5/CHIP association in CHIP mediated cardioprotection is further supported by showing that p90RSK mediated inhibition of ERK5/CHIP axis accelerates cardiac apoptosis after MI, a phenomenon fully reversible by activating ERK5. Two distinct mechanisms have been identified for the action of p90RSK on ERK5/CHIP axis: first, it directly inhibits ERK5/CHIP interaction by competing for binding site on ERK5 and, second, it phosphorylates ERK5 at S496; phospho-S496-ERK5 facilitates angiotensin-II mediated inhibition of CHIP [115].

#### 4.6.2. Availability of Ubiquitination Motifs

Further support for a functional regulation of CHIP mediated ubiquitination was provided by Landrè and coworkers [116]. In the context of IRF-1 (interferon regulatory factor-1) protein stability, they recently reported that both CHIP and MDM2 docked at the Mf2 domain of IRF-1 and subsequent ubiquitination occurred only in the specific lysine residues found predominantly in loop structures that extend from the DNA-binding domain. This E3 docking site is not available when IRF-1 is in its DNA-bound conformation, thus linking IRF-1 function and turnover [116].

## 5. Future Directions

Recent studies provide a plethora of evidences to emphasize the critical role of CHIP in regulating varied biological phenomena. Hence, an obvious question to be addressed is whether and how CHIP itself is regulated. Evidence gathered till date projects CHIP as a highly regulated protein whose function in the cell is critically monitored.

However, we are just beginning to understand various facets of CHIP and several mechanistic questions remain waiting to be answered. For example, we do not understand the mechanisms behind the transcriptional regulation of CHIP, although we do have sufficient published data showing direct relationships between cancers and expression levels of CHIP. Another important issue is substrate specificity. Separating the chaperone dependent and independent activities of CHIP may also be important to explain the occurrence of such a diverse array of its substrates. In this context, is CHIP function regulated through interaction with adaptors and/or modulators? Further, as CHIP is known to catalyse multiple types of polyubiquitination reactions, it would be necessary to have enough knowledge of the various substrates of CHIP being targeted by these different modifications. Also, what are the criteria for selection of E2s that CHIP selects under a particular condition? Finally, a mechanistic understanding of how the intracellular distribution of CHIP is regulated under various physiological conditions will also be critical.

Therefore, understanding how CHIP is regulated in terms of transcriptional, posttranscriptional, posttranslational modifications, and interacting partners and subcellular localization is expected to provide new insights into the overall biological role of CHIP.

## References

[1] Hartl FU, Bracher A, Hayer-Hartl M (2011). Molecular chaperones in protein folding and proteostasis. *Nature*.

[2] Ballinger CA, Connell P, Wu Y (1999). Identification of CHIP, a novel tetratricopeptide repeat-containing protein that interacts with heat shock proteins and negatively regulates chaperone functions. *Molecular and Cellular Biology*.

[3] Kim S-A, Yoon J-H, Kim D-K, Kim S-G, Ahn S-G (2005). CHIP interacts with heat shock factor 1 during heat stress. *FEBS Letters*.

[4] Qian S-B, McDonough H, Boellmann F, Cyr DM, Patterson C (2006). CHIP-mediated stress recovery by sequential ubiquitination of substrates and Hsp70. *Nature*.

[5] McDonough H, Patterson C (2003). CHIP: a link between the chaperone and proteasome systems. *Cell Stress & Chaperones*.

[6] Connell P, Ballinger CA, Jiang J (2001). The co-chaperone CHIP regulates protein triage decisions mediated by heat-shock proteins. *Nature Cell Biology*.

[7] Demand J, Alberti S, Patterson C, Höhfeld J (2001). Cooperation of a ubiquitin domain protein and an E3 ubiquitin ligase during chaperone/proteasome coupling. *Current Biology*.

[8] Jiang J, Ballinger CA, Wu Y (2001). CHIP is a U-box-dependent E3 ubiquitin ligase: identification of Hsc70 as a target for ubiquitylation. *The Journal of Biological Chemistry*.

[9] Mayer RJ, Ciechanover AJ, Rechsteiner M (2008). *Protein Degradation: The Ubiquitin-Proteasome System*.

[10] Meacham GC, Patterson C, Zhang W, Younger JM, Cyr DM (2001). The Hsc70 co-chaperone CHIP targets immature CFTR for proteasomal degradation. *Nature Cell Biology*.

[11] Lamb JR, Tugendreich S, Hieter P (1995). Tetratrico peptide repeat interactions: to TPR or not to TPR?. *Trends in Biochemical Sciences*.

[12] Demand J, Lüders J, Höhfeld J (1998). The carboxy-terminal domain of Hsc70 provides binding sites for a distinct set of chaperone cofactors. *Molecular and Cellular Biology*.

[13] Höhfeld J, Minami Y, Hartl F-U (1995). Hip, a novel cochaperone involved in the eukaryotic Hsc70/Hsp40 reaction cycle. *Cell*.

[14] Ratajczak T, Carello A, Mark PJ (1993). The cyclophilin component of the unactivated estrogen receptor contains a tetratricopeptide repeat domain and shares identity with p59 (FKBP59). *The Journal of Biological Chemistry*.

[15] Wang X, DeFranco DB (2005). Alternative effects of the ubiquitin-proteasome pathway on glucocorticoid receptor down-regulation and transactivation are mediated by CHIP, an E3 ligase. *Molecular Endocrinology*.

[16] Alberti S, Böhse K, Arndt V, Schmitz A, Höhfeld J (2004). The cochaperone HspBP1 inhibits the CHIP ubiquitin ligase and stimulates the maturation of the cystic fibrosis transmembrane conductance regulator. *Molecular Biology of the Cell*.

[17] Murata S, Minami Y, Minami M, Chiba T, Tanaka K (2001). CHIP is a chaperone-dependent E3 ligase that ubiquitylates unfolded protein. *EMBO Reports*.

[18] Dai Q, Zhang C, Wu Y (2003). CHIP activates HSF1 and confers protection against apoptosis and cellular stress. *The EMBO Journal*.

[19] Nikolay R, Wiederkehr T, Rist W, Kramer G, Mayer MP, Bukau B (2004). Dimerization of the human E3 ligase CHIP via a coiled-coil domain is essential for its activity. *The Journal of Biological Chemistry*.

[20] Das AK, Cohen PTW, Barford D (1998). The structure of the tetratricopeptide repeats of protein phosphatase 5: implications for TPR-mediated protein-protein interactions. *The EMBO Journal*.

[21] Sikorski RS, Boguski MS, Goebl M, Hieter P (1990). A repeating amino acid motif in CDC23 defines a family of proteins and a new relationship among genes required for mitosis and RNA synthesis. *Cell*.

[22] Aravind L, Koonin EV (2000). The U box is a modified RING finger—a common domain in ubiquitination. *Current Biology*.

[23] Passmore LA, Barford D (2004). Getting into position: the catalytic mechanisms of protein of protein ubiquitylation. *Biochemical Journal*.

[24] Zhang M, Windheim M, Roe SM (2005). Chaperoned ubiquitylation—crystal structures of the CHIP U box E3 ubiquitin ligase and a CHIP-Ubc13-Uev1a complex. *Molecular Cell*.

[25] Page RC, Pruneda JN, Amick J, Klevit RE, Misra S (2012). Structural insights into the conformation and oligomerization of E2 ubiquitin conjugates. *Biochemistry*.

[26] Xu Z, Kohli E, Devlin KI, Bold M, Nix JC, Misra S (2008). Interactions between the quality control ubiquitin ligase CHIP and ubiquitin conjugating enzymes. *BMC Structural Biology*.

[27] Dikshit P, Jana NR (2007). The co-chaperone CHIP is induced in various stresses and confers protection to cells. *Biochemical and Biophysical Research Communications*.

[28] Stankowski JN, Zeiger SLH, Cohen EL, Defranco DB, Cai J, McLaughlin B (2011). C-terminus of heat shock cognate 70 interacting protein increases following stroke and impairs survival against acute oxidative stress. *Antioxidants & Redox Signaling*.

[29] Jana NR, Dikshit P, Goswami A (2005). Co-chaperone CHIP associates with expanded polyglutamine protein and promotes their degradation by proteasomes. *The Journal of Biological Chemistry*.

[30] Miller VM, Nelson RF, Gouvion CM (2005). CHIP suppresses polyglutamine aggregation and toxicity *in vitro* and *in vivo*. *The Journal of Neuroscience*.

[31] Afrazi A, Sodhi CP, Good M (2012). Intracellular heat shock protein-70 negatively regulates TLR4 signaling in the newborn intestinal epithelium. *The Journal of Immunology*.

[32] Kettern N, Rogon C, Limmer A, Schild H, Höhfeld J (2011). The Hsc/Hsp70 co-chaperone network controls antigen aggregation and presentation during maturation of professional antigen presenting cells. *PLoS ONE*.

[33] Kunisawa J, Shastri N (2006). Hsp90*α* chaperones large C-terminally extended proteolytic intermediates in the MHC class I antigen processing pathway. *Immunity*.

[34] Wang S, Li Y, Hu Y-H (2013). STUB1 is essential for T-cell activation by ubiquitinating CARMA1. *European Journal of Immunology*.

[35] Wei Q, Sha Y, Bhattacharya A (2014). Regulation of IL-4 receptor signaling by STUB1 in lung inflammation. *American Journal of Respiratory and Critical Care Medicine*.

[36] Yang M, Wang C, Zhu X (2011). E3 ubiquitin ligase CHIP facilitates Toll-like receptor signaling by recruiting and polyubiquitinating Src and atypical PKC*ζ*. *Journal of Experimental Medicine*.

[37] Meng Y, Chen C, Wang L (2013). Toll-like receptor-2 ligand peptidoglycan upregulates expression and ubiquitin ligase activity of CHIP through JNK pathway. *Cellular Physiology and Biochemistry*.

[38] Patani N, Jiang W, Newbold R, Mokbel K (2010). Prognostic implications of carboxyl-terminus of Hsc70 interacting protein and lysyl-oxidase expression in human breast cancer. *Journal of Carcinogenesis*.

[39] Kajiro M, Hirota R, Nakajima Y (2009). The ubiquitin ligase CHIP acts as an upstream regulator of oncogenic pathways. *Nature Cell Biology*.

[40] Ruckova E, Muller P, Nenutil R, Vojtesek B (2012). Alterations of the Hsp70/Hsp90 chaperone and the HOP/CHIP co-chaperone system in cancer. *Cellular & Molecular Biology Letters*.

[41] Wang Y, Ren F, Wang Y (2013). CHIP/Stub1 functions as a tumor suppressor and represses NF-*κ*B-mediated signaling in colorectal cancer. *Carcinogenesis*.

[42] Gan L, Liu D-B, Lu H-F (2012). Decreased expression of the carboxyl terminus of heat shock cognate 70 interacting protein in human gastric cancer and its clinical significance. *Oncology Reports*.

[43] Guo J, Ren F, Wang Y (2012). miR-764-5p promotes osteoblast differentiation through inhibition of CHIP/STUB1 expression. *Journal of Bone and Mineral Research*.

[44] Petroski MD, Deshaies RJ (2005). Function and regulation of cullin-RING ubiquitin ligases. *Nature Reviews Molecular Cell Biology*.

[45] de Bie P, Ciechanover A (2011). Ubiquitination of E3 ligases: self-regulation of the ubiquitin system via proteolytic and non-proteolytic mechanisms. *Cell Death & Differentiation*.

[46] Fallon L, Bélanger CML, Corera AT (2006). A regulated interaction with the UIM protein Eps15 implicates Parkin in EGF receptor trafficking and PI(3)K-Akt signalling. *Nature Cell Biology*.

[47] Imai Y, Soda M, Hatakeyama S (2002). CHIP is associated with Parkin, a gene responsible for familial Parkinson’s disease, and enhances its ubiquitin ligase activity. *Molecular Cell*.

[48] Lees MJ, Peet DJ, Whitelaw ML (2003). Defining the role for XAP2 in stabilization of the dioxin receptor. *The Journal of Biological Chemistry*.

[49] Kim HT, Kim KP, Uchiki T, Gygi SP, Goldberg AL (2009). S5a promotes protein degradation by blocking synthesis of nondegradable forked ubiquitin chains. *The EMBO Journal*.

[50] Sisoula C, Gonos ES (2011). CHIP E3 ligase regulates mammalian senescence by modulating the levels of oxidized proteins. *Mechanisms of Ageing and Development*.

[51] Scaglione KM, Zavodszky E, Todi SV (2011). Ube2w and ataxin-3 coordinately regulate the ubiquitin ligase CHIP. *Molecular Cell*.

[52] Dephoure N, Zhou C, Villén J (2008). A quantitative atlas of mitotic phosphorylation. *Proceedings of the National Academy of Sciences of the United States of America*.

[53] Sengupta S, Badhwar I, Upadhyay M, Singh S, Ganesh S (2011). Malin and laforin are essential components of a protein complex that protects cells from thermal stress. *Journal of Cell Science*.

[54] Lim MK, Kawamura T, Ohsawa Y (2007). Parkin interacts with LIM Kinase 1 and reduces its cofilin-phosphorylation activity via ubiquitination. *Experimental Cell Research*.

[55] Woo C-H, Le N-T, Shishido T (2010). Novel role of C terminus of Hsc70-interacting protein (CHIP) ubiquitin ligase on inhibiting cardiac apoptosis and dysfunction via regulating ERK5-mediated degradation of inducible cAMP early repressor. *FASEB Journal*.

[56] Millan ICR, Squillace ALA, Gava LM, Ramosa CHI (2012). The stability of wild-type and deletion mutants of human C-terminus Hsp70-interacting protein (CHIP). *Protein & Peptide Letters*.

[57] Fujii T, Machino K, Andoh H, Satoh T, Kondo Y (1990). Calcium-dependent control of caldesmon-actin interaction by S100 protein. *Journal of Biochemistry*.

[58] Heierhorst J, Kobe B, Feil SC (1996). Ca^2+^/S100 regulation of giant protein kinases. *Nature*.

[59] Shimamoto S, Kubota Y, Yamaguchi F, Tokumitsu H, Kobayashi R (2013). Ca^2+^/S100 proteins act as upstream regulators of the chaperone-associated ubiquitin ligase CHIP (C terminus of Hsc70-interacting protein). *The Journal of Biological Chemistry*.

[60] Kazlauskas A, Poellinger L, Pongratz I (2000). The immunophilin-like protein XAP2 regulates ubiquitination and subcellular localization of the dioxin receptor. *Journal of Biological Chemistry*.

[61] Kuzhandaivelu N, Cong Y-S, Inouye C, Yang W-M, Seto E (1996). XAP2, a novel hepatitis B virus X-associated protein that inhibits X transactivation. *Nucleic Acids Research*.

[62] Meyer BK, Petrulis JR, Perdew GH (2000). Aryl hydrocarbon (Ah) receptor levels are selectively modulated by hsp90-associated immunophilin homolog XAP2. *Cell Stress & Chaperones*.

[63] Koller-Eichhorn R, Marquardt T, Gail R (2007). Human OLA1 defines an ATPase subfamily in the Obg family of GTP-binding proteins. *The Journal of Biological Chemistry*.

[64] Mao R-F, Rubio V, Chen H, Bai L, Mansour OC, Shi Z-Z (2013). OLA1 protects cells in heat shock by stabilizing HSP70. *Cell Death & Disease*.

[65] Kabani M, McLellan C, Raynes DA, Guerriero V, Brodsky JL (2002). HspBP1, a homologue of the yeast Fes1 and Sls1 proteins, is an Hsc70 nucleotide exchange factor. *FEBS Letters*.

[66] Takayama S, Bimston DN, Matsuzawa S-I (1997). BAG-1 modulates the chaperone activity of Hsp70/Hsc70. *The EMBO Journal*.

[67] Takayama S, Sato T, Krajewski S (1995). Cloning and functional analysis of BAG-1: a novel Bcl-2-binding protein with anti-cell death activity. *Cell*.

[68] Takayama S, Xie Z, Reed JC (1999). An evolutionarily conserved family of Hsp70/Hsc70 molecular chaperone regulators. *The Journal of Biological Chemistry*.

[69] Brive L, Takayama S, Briknarová K (2001). The carboxyl-terminal lobe of Hsc70 ATPase domain is sufficient for binding to BAG1. *Biochemical and Biophysical Research Communications*.

[70] Alberti S, Demand J, Esser C, Emmerich N, Schild H, Höhfeld J (2002). Ubiquitylation of BAG-1 suggests a novel regulatory mechanism during the sorting of chaperone substrates to the proteasome. *The Journal of Biological Chemistry*.

[71] Höhfeld J, Jentsch S (1997). GrpE-like regulation of the Hsc70 chaperone by the anti-apoptotic protein BAG-1. *The EMBO Journal*.

[72] Sondermann H, Scheufler C, Schneider C, Höhfeld J, Hartl F-U, Moarefi I (2001). Structure of a Bag/Hsc70 complex: convergent functional evolution of Hsp70 nucleotide exchange factors. *Science*.

[73] Lüders J, Demand J, Höhfeld J (2000). The ubiquitin-related BAG-1 provides a link between the molecular chaperones Hsc70/Hsp70 and the proteasome. *The Journal of Biological Chemistry*.

[74] Arndt V, Daniel C, Nastainczyk W, Alberti S, Höhfeld J (2005). BAG-2 acts as an inhibitor of the chaperone-associated ubiquitin ligase CHIP. *Molecular Biology of the Cell*.

[75] Dai Q, Qian S-B, Li H-H (2005). Regulation of the cytoplasmic quality control protein degradation pathway by BAG2. *The Journal of Biological Chemistry*.

[76] Arndt V, Dick N, Tawo R (2010). Chaperone-assisted selective autophagy is essential for muscle maintenance. *Current Biology*.

[77] Behl C (2011). BAG3 and friends: co-chaperones in selective autophagy during aging and disease. *Autophagy*.

[78] Kalia LV, Kalia SK, Chau H, Lozano AM, Hyman BT, McLean PJ (2011). Ubiquitinylation of *α*-synuclein by carboxyl terminus hsp70-interacting protein (CHIP) is regulated by bcl-2-associated athanogene 5 (BAG5). *PLoS ONE*.

[79] Chapple JP, van der Spuy J, Poopalasundaram S, Cheetham ME (2004). Neuronal DnaJ proteins HSJ1a and HSJ1b: a role in linking the Hsp70 chaperone machine to the ubiquitin-proteasome system?. *Biochemical Society Transactions*.

[80] Cheetham ME, Jackson AP, Anderton BH (1994). Regulation of 70-kDa heat-shock-protein ATPase activity and substrate binding by human DnaJ-like proteins, HSJ1a and HSJ1b. *European Journal of Biochemistry*.

[81] Westhoff B, Chapple JP, van der Spuy J, Höhfeld J, Cheetham ME (2005). HSJ1 is a neuronal shuttling factor for the sorting of chaperone clients to the proteasome. *Current Biology*.

[82] Hyoung TK, Kwang PK, Lledias F (2007). Certain pairs of ubiquitin-conjugating enzymes (E2s) and ubiquitin-protein ligases (E3s) synthesize nondegradable forked ubiquitin chains containing all possible isopeptide linkages. *The Journal of Biological Chemistry*.

[83] Rubin DM, van Nocker S, Glickman M (1997). ATPase and ubiquitin-binding proteins of the yeast proteasome. *Molecular Biology Reports*.

[84] Burnett B, Li F, Pittman RN (2003). The polyglutamine neurodegenerative protein ataxin-3 binds polyubiquitylated proteins and has ubiquitin protease activity. *Human Molecular Genetics*.

[85] Winborn BJ, Travis SM, Todi SV (2008). The deubiquitinating enzyme ataxin-3, a polyglutamine disease protein, edits Lys63 linkages in mixed linkage ubiquitin chains. *The Journal of Biological Chemistry*.

[86] Ventii KH, Wilkinson KD (2008). Protein partners of deubiquitinating enzymes. *Biochemical Journal*.

[87] Kuhlbrodt K, Janiesch PC, Kevei É, Segref A, Barikbin R, Hoppe T (2011). The Machado-Joseph disease deubiquitylase ATX-3 couples longevity and proteostasis. *Nature Cell Biology*.

[88] Zhong X, Pittman RN (2006). Ataxin-3 binds VCP/p97 and regulates retrotranslocation of ERAD substrates. *Human Molecular Genetics*.

[89] Kitada T, Asakawa S, Hattori N (1998). Mutations in the Parkin gene cause autosomal recessive Juvenile Parkinsonism. *Nature*.

[90] Feany MB, Pallanck LJ (2003). Parkin: a multipurpose neuroprotective agent?. *Neuron*.

[91] Takahashi R, Imai Y (2003). Pael receptor, endoplasmic reticulum stress, and Parkinson’s disease. *Journal of Neurology*.

[92] Imai J, Yashiroda H, Maruya M, Yahara I, Tanaka K (2003). Proteasomes and molecular chaperones: cellular machinery responsible for folding and destruction of unfolded proteins. *Cell Cycle*.

[93] Solano RM, Casarejos MJ, Gómez A, Perucho J, de Yébenes JG, Mena MA (2012). Parkin null cortical neuronal/glial cultures are resistant to amyloid-*β*1-42 toxicity: a role for autophagy?. *Journal of Alzheimer's Disease*.

[94] Amati B, Vlach J (1999). Kip1 meets SKP2: new links in cell-cycle control. *Nature cell biology*.

[95] Huang Z, Nie L, Xu M, Sun X-H (2004). Notch-induced E2A degradation requires CHIP and Hsc70 as novel facilitators of ubiquitination. *Molecular and Cellular Biology*.

[96] Kim SY, Herbst A, Tworkowski KA, Salghetti SE, Tansey WP (2003). Skp2 regulates Myc protein stability and activity. *Molecular Cell*.

[97] Nie L, Wu H, Sun X-H (2008). Ubiquitination and degradation of Tal1/SCL are induced by notch signaling and depend on Skp2 and CHIP. *The Journal of Biological Chemistry*.

[98] Nie L, Xu M, Vladimirova A, Sun X-H (2003). Notch-induced E2A ubiquitination and degradation are controlled by MAP kinase activities. *The EMBO Journal*.

[99] Paul I, Ahmed SF, Bhowmik A, Deb S, Ghosh MK (2013). The ubiquitin ligase CHIP regulates c-Myc stability and transcriptional activity. *Oncogene*.

[100] Anderson LG, Meeker RB, Poulton WE, Huang DY (2010). Brain distribution of carboxy terminus of Hsc70-interacting protein (CHIP) and its nuclear translocation in cultured cortical neurons following heat stress or oxygen-glucose deprivation. *Cell Stress & Chaperones*.

[101] Adachi H, Waza M, Tokui K (2007). CHIP overexpression reduces mutant androgen receptor protein and ameliorates phenotypes of the spinal and bulbar muscular atrophy transgenic mouse model. *The Journal of Neuroscience*.

[102] Al-Ramahi I, Lam YC, Chen H-K (2006). CHIP protects from the neurotoxicity of expanded and wild-type ataxin-1 and promotes their ubiquitination and degradation. *The Journal of Biological Chemistry*.

[103] Choi JY, Ryu JH, Kim H-S (2007). Co-chaperone CHIP promotes aggregation of ataxin-1. *Molecular and Cellular Neuroscience*.

[104] Wang S, Wu X, Zhang J (2012). CHIP functions as a novel suppressor of tumour angiogenesis with prognostic significance in human gastric cancer. *Gut*.

[105] Shang Y, Zhao X, Xu X (2009). CHIP functions an E3 ubiquitin ligase of Runx1. *Biochemical and Biophysical Research Communications*.

[106] Li X, Huang M, Zheng H (2008). CHIP promotes Runx2 degradation and negatively regulates osteoblast differentiation. *Journal of Cell Biology*.

[107] Tripathi V, Ali A, Bhat R, Pati U (2007). CHIP chaperones wild type p53 tumor suppressor protein. *The Journal of Biological Chemistry*.

[108] Grove DE, Rosser MFN, Hong YR, Naren AP, Cyr DM (2009). Mechanisms for rescue of correctable folding defects in CFTRΔF508. *Molecular Biology of the Cell*.

[109] Slotman JA, Almeida ACS, Hassink GC (2012). Ubc13 and COOH terminus of Hsp70-interacting protein (CHIP) are required for growth hormone receptor endocytosis. *The Journal of Biological Chemistry*.

[110] Sakagami Y, Kudo T, Tanimukai H (2013). Involvement of endoplasmic reticulum stress in tauopathy. *Biochemical and Biophysical Research Communications*.

[111] Lee H, Sengupta N, Villagra A, Rezai-Zadeh N, Seto E (2006). Histone deacetylase 8 safeguards the human ever-shorter telomeres 1B (hEST1B) protein from ubiquitin-mediated degradation. *Molecular and Cellular Biology*.

[112] DaSilva J, Gioeli D, Weber MJ, Parsons SJ (2009). The neuroendocrine-derived peptide parathyroid hormone-related protein promotes prostate cancer cell growth by stabilizing the androgen receptor. *Cancer Research*.

[113] Xiao L, Chen D, Hu P (2011). The c-Abl-MST1 signaling pathway mediates oxidative stress-induced neuronal cell death. *The Journal of Neuroscience*.

[114] Wang Y, Guan S, Acharya P (2011). Ubiquitin-dependent proteasomal degradation of human liver cytochrome P450 2E1: identification of sites targeted for phosphorylation and ubiquitination. *The Journal of Biological Chemistry*.

[115] Le N-T, Takei Y, Shishido T (2012). P90RSK targets the ERK5-CHIP ubiquitin E3 ligase activity in diabetic hearts and promotes cardiac apoptosis and dysfunction. *Circulation Research*.

[116] Landré V, Pion E, Narayan V, Xirodimas DP, Ball KL (2013). DNA-binding regulates site-specific ubiquitination of IRF-1. *Biochemical Journal*.

